# N‐methylation of histidine to tune tautomeric preferences in histidine‐heme coordination and enzyme‐mimetic catalysis

**DOI:** 10.1002/smo.20240012

**Published:** 2024-07-18

**Authors:** Ruikai Du, Yunbo Lv, Haifeng Wu, Baoli Zhang, Yuanxi Liu, Shichao Xu, Shan Li, Zhen‐Gang Wang

**Affiliations:** ^1^ State Key Laboratory of Organic−Inorganic Composites Key Lab of Biomedical Materials of Natural Macromolecules (Ministry of Education) Beijing Laboratory of Biomedical Materials College of Materials Science and Engineering Beijing University of Chemical Technology Beijing China

**Keywords:** heme‐dependent enzyme, histidine, self‐assembly, tautomeric preference

## Abstract

Enzymes with active sites involving histidine selectively utilize either the δ‐ or ε‐nitrogen atom (N_δ_ or N_ε_) of the histidine imidazole for catalysis. However, evaluating the impact of N_δ_ and N_ε_ is difficult, and directly integrating noncanonical N‐methylated histidine within enzymes poses risks due to laborious procedures. In this study, we present the self‐assembly of Fmoc‐Histidine (Fmoc‐His) with hemin to create a peroxidase‐mimetic catalyst, in which either the N_ε_ or N_δ_ of histidine is methylated to modify the tautomeric preferences, thereby tuning hemin catalysis. UV‐vis spectra, ^1^H‐NMR, and fluorescence experiments elucidate that the N‐methylation of histidine alters the self‐assembly propensity of Fmoc‐His, and affects the binding affinity of histidine to hemin iron, with Fmoc‐_δm_His/hemin exhibiting stronger binding than Fmoc‐_εm_His/hemin. Theoretical simulation results suggest that _εm_His and _δm_His ligation produce a saddled structure and planar structure of hemin, respectively, stemming from the disparity of steric hindrance at the N_ε_ and N_δ_ positions. The significant inhibition of hemin's oxidative activity by Fmoc‐_δm_His is observed, likely due to the strong binding of Fmoc‐_δm_His, potentially hindering access of the substrate, H_2_O_2_, to the hemin iron. Conversely, Fmoc‐_εm_His enhances hemin catalysis, surpassing even Fmoc‐His alone. This differential impact of Fmoc‐_εm_His and Fmoc‐_δm_His on hemin activity is further corroborated by apparent activation energy and kinetic parameters (*k*_cat_, *k*_cat_/*K*_m_). This study sheds light on the heterogeneous biological effects at the nitrogen positions of histidine imidazole and offers insights into designing supramolecular metalloenzymes.

## Graphical abstract

This work presents the self‐assembly of Fmoc‐Histidine with hemin to create a supramolecular peroxidase‐mimetic catalyst. By metylating either the N_ε_ or N_δ_ of histidine, the tautomeric preferences of histidine and its binding affinity to hemin are adjusted, affecting substrate access to hemin and catalytic activity of hemin.

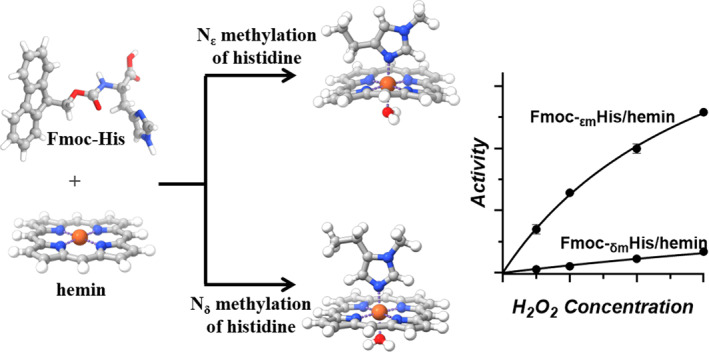

## 1 INTRODUCTION

In the complex interplay of biological systems, enzymes assume the role of primary orchestrators, regulating and catalyzing reactions pivotal to the sustenance of life. The precise spatial arrangement of key amino acid residues exerts profound influence over the specificity and efficiency of biochemical transformations. A variety of approaches, including site‐directed mutagenesis, rational design, or directed evolution, were developed to modify the key residues to engineer the catalytic functions of enzymes or to unravel the mechanisms behind the biological processes. However, these approaches are plagued by laborious screening, off‐target mutations, and occasional alterations in protein folding structures.^[1]^ Supramolecular catalysts, created by assembling catalytic species, have attracted considerable attention for catalyzing reactions similar to those catalyzed by natural enzymes.^[2]^ These catalysts possess simpler structures compared to native enzymes, enabling customization of catalytic functions through modifications of the catalytic species (also referred to as molecular building blocks). This facilitates the exploration of the catalytic effects of essential residues within a straightforward synthetic framework.

**FIGURE 1:**
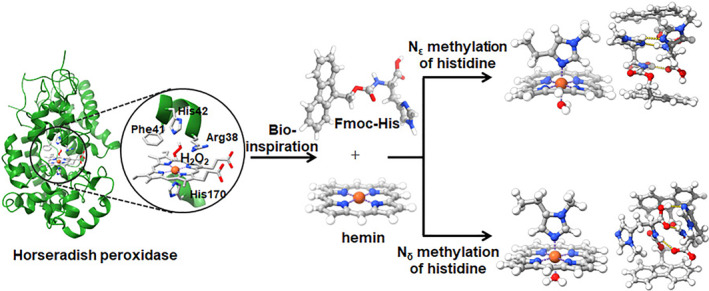
Inspired by the catalytic active site in natural HRP (PDB: 1W4W), fluorenyl‐modified histidine (or its N‐methylated tautomer) is self‐assembled with hemin to create a peroxidase‐mimetic supramolecular catalyst. The histidine‐hemin interaction and the activity can be affected by the methylation at either δ or ε‐position of histidine imidazole. In the chemical structures, nitrogen atoms are depicted in blue, oxygen atoms in red, iron atoms in brown, carbon atoms in dark gray, and hydrogen atoms in light gray.

Histidine has been employed by biological systems as an essential component, owing to the unique prowess of its side chain imidazole, which facilitates proton transfer, metal coordination, and substrate binding, imbuing enzyme active sites with a remarkable degree of flexibility and adaptability. The imidazole of histidine has two nitrogen atoms, ε‐nitrogen (N_ε_) and δ‐nitrogen (N_δ_), which possess electronic and steric differences. Copper nitrite reductase (CuNiR) and peptidylglycine α‐hydroxylating monooxygenase (PHM), which both feature a type 2 Cu center with a His3 coordination sphere, demonstrate distinct functions due to disparities in histidine ligation, with PHM binding copper via the N_δ_ atoms of histidine residues, whereas CuNiR binds through the N_ε_ atoms.^[3]^ Actually, a myriad of metalloenzymes, including catechol oxidase, peroxidases, carbonic anhydrases, use the specific nitrogens of the imidazole in histidine for direct catalysis or ligation with the metal center (Figure S1) based on factors such as steric hindrance, electronic properties, and the specific requirements of the catalytic mechanism. Comparing N_δ_ and N_ε_ atoms directly is difficult, yet this complexity may be circumvented by using the noncanonical amino acids _δm_His and _εm_His (histidine methylated at the δ‐ or ε‐nitrogen, respectively). N‐methylated histidines have contributed to the functions of natural enzymatic active sites across diverse biological processes, including signaling, gene regulation, and metabolism. Some efforts have also been reported in the incorporation of the N‐methylated histidine in enzyme design, such as hydrolase.^[4]^ However, such operations are subject to the challenges in designing biosynthetic pathways, expanding the genetic code, ensuring selective incorporation, and maintaining protein structure. Herein, we reported the self‐assembly of fluorenyl‐modified histidine (or its N‐methylated tautomer) with hemin to create a peroxidase‐mimetic supramolecular catalyst, in which the fluorenyl moiety stacked to locate the histidine residues around hemin (Figure 1). The histidine residues can stay proximal to hemin to act like a ligand, and at the distal side of hemin to promote the H_2_O_2_ adsorption to hemin, facilitating the formation of the reactive intermediate Compound I.[^2^, ^5^] The N‐methylation of histidine at different positions significantly altered the coordination affinity of hemin to histidine, as well as the assembly propensity of the fluorenyl‐histidine. Interestingly, this resulted in the enhancement or the complete suppression of the hemin activity. These results may shed light on the heterogeneity of the nitrogen of histidine imidazole during the catalysis of the heme‐dependent enzymes, and also provideavenues for engineering the catalytic function of native enzymes and the synthetic catalysts via the modification of the essential residues.

## 2 RESULTS AND DISCUSSION

**FIGURE 2:**
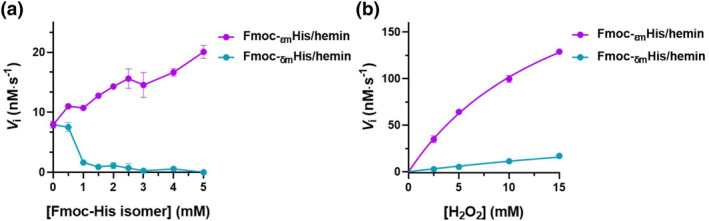
(a) Initial velocity of catalytic oxidation of TMB by Fmoc‐_εm_His/hemin and Fmoc‐_δm_His/hemin assemblies. [hemin] = 0.5 μM, [TMB] = 0.3 mM, [H_2_O_2_] = 1 mM. (b) The initial velocity of catalytic oxidation of TMB by Fmoc‐_εm_His/hemin and Fmoc‐_δm_His/hemin assemblies at different H_2_O_2_ concentrations. [Fmoc‐_εm_His] = 3 mM, [Fmoc‐_δm_His] = 3 mM, [hemin] = 0.5 μM, [TMB] = 0.3 mM.

With H_2_O_2_ and 3,3′,5,5′‐tetramethylbenzidine (TMB) as the substrates, we examined the catalytic activities of Fmoc‐His/hemin, Fmoc‐_εm_His/hemin, and Fmoc‐_δm_His/hemin assemblies, by monitoring the time‐dependent absorbance changes at 652 nm, allowing us to compare the impact of histidine methylation sites on the peroxidase‐mimetic activity of hemin. Figure S2 indicates that the activity of Fmoc‐His/hemin showed dependence on the concentration of Fmoc‐His, suggesting that the coordination of histidine can enhance hemin activity. Fmoc‐_εm_His/hemin showed a comparable hemin activity to Fmoc‐His, while Fmoc‐_δm_His seemed to impede hemin catalysis, especially at higher concentrations (dropped to zero at 5 mM) (Figure 2a). When the concentration of the amphiphilic ligands exceeded 5 mM, Fmoc‐His/hemin exhibited a notable decrease, indicating the superiority of Fmoc‐_εm_His over Fmoc‐His in promoting the formation of the catalyst. We then used the Michaelis‐Menten fitting approach to evaluate the apparent kinetic parameters of turnover number (*k*_cat_) and catalytic efficiency (*k*_cat_/*K*_m_) of Fmoc‐_εm_His/hemin and Fmoc‐_δm_His/hemin (Figure 2b, Figures S3 and S4). These kinetic values of Fmoc‐_εm_His/hemin, with respect to H_2_O_2_ reduction or TMB oxidation, were all significantly higher than those of Fmoc‐_δm_His/hemin (Table 1). Table S1 shows the kinetic parameters of our hemin‐contained catalysts and some of the reported peroxidase mimics. Our catalysts only displayed moderate *k*_cat_/*K*_m_ values, but for the first time exhibited a dependence on the methylation of the histidine ligands. The effect of histidine methylation on the catalytic activities of the hemin complexes was also assessed through the calculation of apparent activation energies (*E*_a_) using the Arrhenius equation (Figure S5). The results demonstrate that the activation energy (*E*_a_) value 14.23 kJ·mol^−1^ for Fmoc‐_εm_His/hemin was significantly lower than that of Fmoc‐_δm_His/hemin 117.04 kJ·mol^−1^, suggesting a diminished barrier for the activation of hemin by H_2_O_2_ to form the transition states. In addition to TMB as the reductive substrate, Fmoc‐_εm_His/hemin also exhibited significantly higher activity towards oxidation of the 2,4‐dicholorophenyl (2,4‐DCP) (with 4‐aminopyrine as the chromogenic agent) (Figure S6A) or 2,2‐diazine‐bis(3‐ethyl‐benzothiazole‐6‐sulfonic acid) diammonium salt (ABTS) (Figure S6B), which have distinct molecular structures from TMB. These results confirmed that methylation of histidine at the ε‐nitrogen site favored hemin catalysis over methylation at the δ‐nitrogen site.

**TABLE 1:** Apparent kinetic parameters for the hemin complexes with respect to H_2_O_2_ reduction and TMB oxidation.

Samples	Fmoc‐_εm_His/hemin	Fmoc‐_δm_His/hemin
*K*_m_ (H_2_O_2_) (mM)	16.08	64.30
*k*_cat_ (H_2_O_2_) (s^−1^)	0.5306	0.167
*k*_cat_/*K*_m_ (H_2_O_2_) (s^−1^ M^−1^)	32.99	2.59
*K*_m_ (TMB) (mM)	0.1449	0.04965
*k*_cat_ (TMB) (s^−1^)	0.661	0.048
*k*_cat_/*K*_m_ (TMB) (s^−1^ M^−1^)	4561	967.7

**FIGURE 3:**
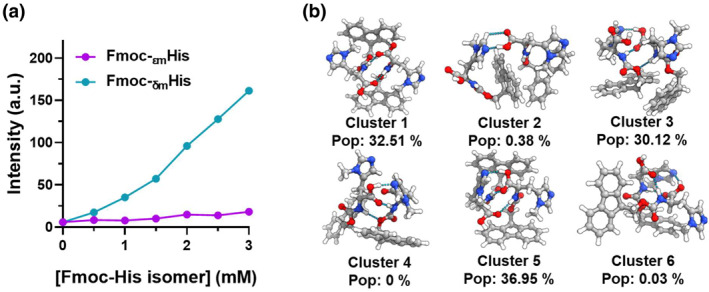
(a) Fluorescence intensity of thioflavin T binding to Fmoc‐_εm_His and Fmoc‐_δm_His, respectively. [ThT] = 100 μM. (b) Schematic diagram of the spatial location of the Fmoc‐_δm_His dimers. In the chemical structures, nitrogen atoms are depicted in blue, oxygen atoms in red, carbon atoms in dark gray, and hydrogen atoms in light gray.

To understand the mechanism behind the methylation effect of histidine, we investigated the structures of the self‐assemblies and the coordination environment of hemin. The fluorescence of pyrene (the intensity ratio of the first and third vibronic peaks, I_1_/I_3_) was used to probe the polarity of the microenvironments within the self‐assembled complexes (Figure S7).^[6]^ The I_1_/I_3_ values of pyrene in Fmoc‐_δm_His were lower than that in Fmoc‐_εm_His, reflecting the greater hydrophobic association of the fluorenyl moiety. Fluorescence spectra confirmed the existence of parallel stacking of the fluorenyl moiety of Fmoc‐_δm_His and Fmoc‐_εm_His, as evidenced by the emission at 460 nm (Figure S8).^[7]^ Then, we used thioflavin T (ThT) as the probe, known to interact with amyloid by stacking and aligning, resulting in a significant enhancement in fluorescence emission at 490 nm (Figure S9 and Figure 3a).^[8]^ The results indicated that upon binding to Fmoc‐_δm_His, the fluorescence intensity of ThT underwent a significantly greater enhancement compared to its binding to Fmoc‐_εm_His, confirming the stronger self‐assembly propensity of Fmoc‐_δm_His than Fmoc‐_εm_His. Using conformational search of dimers, we found that compared to Fmoc‐_εm_His, Fmoc‐_δm_His has greater possibility to form aggregates driven by intermolecular hydrogen bonding (Figure S10 and Figure 3b). The fluorescence and the theoretical simulation results suggest that methylation at the δ‐nitrogen position of histidine promotes the self‐assembly of Fmoc‐His more effectively than methylation at the ε‐nitrogen position.

**FIGURE 4:**
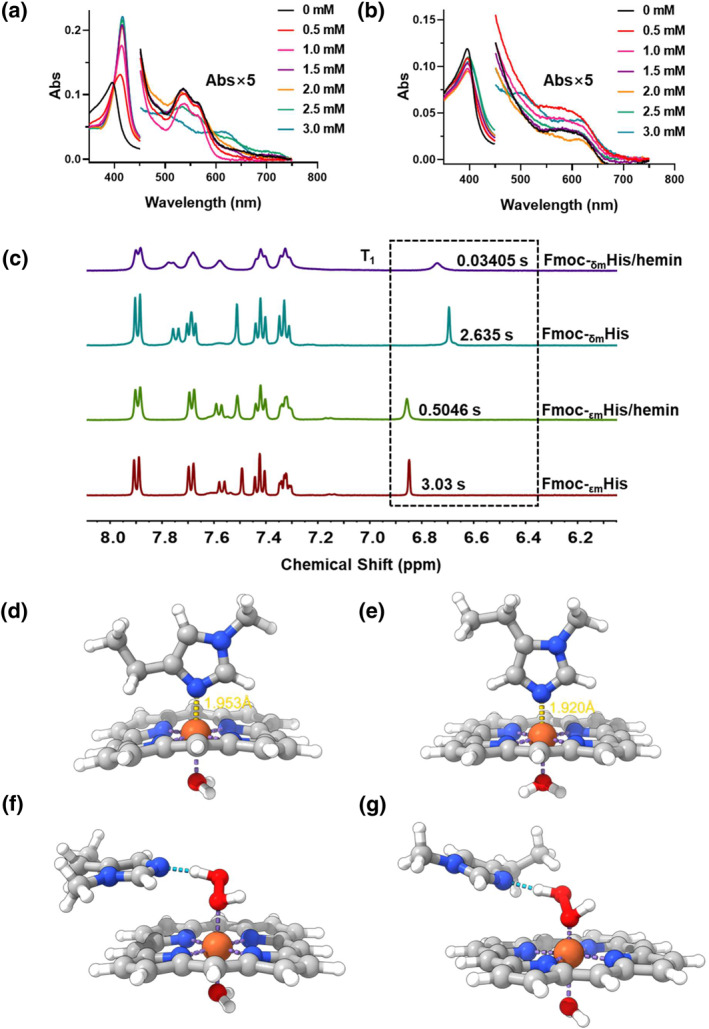
UV‐Vis spectra of (a) Fmoc‐_δm_His/hemin and (b) Fmoc‐_εm_His/hemin. [hemin] = 2 μM. (c) ^1^H NMR spectra of fluorenyl‐modified histidine derivatives and fluorenyl‐modified histidine derivatives/hemin in DMSO‐*d*_6_. Theoretical model of the coordination of (d) _εm_His and (e) _δm_His with hemin iron, respectively. In the chemical structures, nitrogen atoms are depicted in blue, oxygen atoms in red, iron atoms in brown, carbon atoms in dark gray, and hydrogen atoms in light gray. Theoretical models of (f) Fmoc‐_δm_His/hemin and (g) Fmoc‐_εm_His/hemin adsorption with H_2_O_2_, respectively. In the chemical structure, the nitrogen atom is represented by blue, the oxygen atom by red, the iron atom by brown, the carbon atom by dark gray, and the hydrogen atom by light gray.

UV–Vis spectroscopy was employed to study the coordination state of hemin. The free hemin displayed bands a Soret peak at 395 nm, a shoulder at 365 nm, and a low‐intensity band at 498 and 615 nm (Q band), indicating the presence of a mixture of the μ‐oxo bihemin (the dimer of hemin) along with the monomeric hemin hydroxide (hematin).^[9]^ In the presence of Fmoc‐_δm_His in the range of 0–3 mM, the Soret peak of hemin experienced an evident redshift from 395 to 415 nm, accompanied by the appearance of two bands at 535 and 562 nm (Figure 4a). These spectral features are attributed to the charge‐transfer transition from imidazole to iron and the presence of a low‐spin, six‐coordinated species with two strong‐field ligands, one of which likely corresponds to a histidine residue on the sixth coordination site.^[10]^ In comparison, Fmoc‐_εm_His had a much less significant impact on the environment of hemin, indicating the moderate coordination of ligands to hemin (Figure 4b). Additionally, hydrogen nuclear magnetic resonance (^1^H‐NMR) was employed to explore the interaction between Fmoc‐_εm_His or Fmoc‐_δm_His to hemin iron, which can accelerate the relaxation of ^1^H nuclei nearby, resulting in broadening of the corresponding resonance lines.^[11]^ Figure 4c shows that as the ratio of hemin to the histidine increased, there was more significant signal broadening of hydrogen attached to imidazole C2 and C5 of Fmoc‐_δm_His than Fmoc‐_εm_His. The longitudinal relaxation time (T_1_) for Fmoc‐_δm_His changed from 2.635 to 0.03405 s after coordination to hemin, whereas for Fmoc‐_δm_His, it changed from 3.03 to 0.5046 s. These data support a more efficient coordination of Fmoc‐_δm_His to hemin than Fmoc‐_εm_His.

The disparity in the coordination of these two methylated histidines to hemin iron, as well as the resultant oxidative activity, may be attributed to the following factors. Firstly, the density functional theory (DFT) calculations indicate that when Hie and _εm_His are bound to the hemin surface, the porphyrin structure undergoes a saddle‐shaped distortion due to the proximity of the amino acid backbones to the porphyrin (Figure S11 and Figure 4d). However, this distortion is not observed when porphyrin is in the presence of Hid and _δm_His (Figure S12 and Figure 4e). This distortion results in a stretching of the distance between the coordinated N atoms and iron by approximately 0.03–0.04 Å. Additionally, the relative energy of the distorted porphyrin ring is 3.2–3.3 kcal/mol higher than that of the planar conformation. The elongated N‐Fe bonds and the relatively higher energy of the porphyrin ring might enhance the accessibility of H_2_O_2_ to the distal side of hemin, a crucial step in the formation of the reaction intermediate Compound I in peroxidative catalysis of hemin.^[12]^ The DFT calculations indicate that the adsorption energy of H_2_O_2_ on Fmoc‐_δm_His/hemin (Figure 4f) and Fmoc‐_εm_His/hemin (Figure 4g) is −23.81 kcal/mol and −28.82 kcal/mol, respectively, confirming the easity adsorption of H_2_O_2_ to Fmoc‐_εm_His/hemin. Furthermore, based on the structures of the histidine dimers (Figure 3b and Figure S10), there appears to be a greater amount of imidazole present in Fmoc‐_δm_His dimers that do not engage in hydrogen bonds, as opposed to Fmoc‐_εm_His, which might also contribute to the enhanced coordination of Fmoc‐_δm_His to hemin. Second, the stronger association of Fmoc‐_δm_His compared to Fmoc‐_εm_His, as evidenced by fluorescence experiments (Figures S7 and S9 and Figure 4a), suggests a higher concentration of the imidazole ligand around hemin within the Fmoc‐_δm_His assembly, implying a greater potential for _δm_His to bind to hemin at the distal side. This may also contribute to the observed inhibition of the peroxidative activity of the Fmoc‐_δm_His/hemin catalyst. Third, it is known that histidine (His42) located on the distal side of the heme in native horseradish peroxidase can serve as a base catalyst to deprotonate H_2_O_2_ that adsorbed at the heme iron during catalysis.^[2b]^ One may concern the disparity between pKa of the imidazole nitrogen of _εm_His (5.91) and _δm_His (6.52), which can cause their distinct ability to be protonated.^[13]^ However, the single point energy, calculated via DFT at the M06‐2X‐D3/def2‐TZVP level, reveals Hid (−14.41 kcal/mol) and Hie (−14.48 kcal/mol) exhibited remarkably similar proton binding free energies (Figure S13), along with potential minimum values (−56.93 kcal/mol and −54.83 kcal/mol) (Figure S14), the latter discerned through quantitative surface electrostatic potential analysis.^[14]^ These similarities were also extended to methylated histidine variants (Figures S13 and S14). These results suggest that various nitrogens within the histidine imidazole, even when methylated, exhibit nearly identical tendencies to deprotonate H_2_O_2_. It is interesting to find that the catalytic activity of Fmoc‐_δm_His was accelerated significantly as the reaction temperture was elevated, while that of Fmoc‐_εm_His had a slight increase (Figure S15). This is attributed to the stronger coordination of _δm_His to hemin than that of _εm_His to hemin, leading to a more stable Fmoc‐_δm_His/hemin complex.

In hemin complexes containing Fmoc‐His or its methylation variants, the imidazole may be situated both at the proximal and distal side of hemin, acting as a ligand and an acid/base catalyst. It was reported by our group that the peptides can self‐assemble with G‐quadruplex DNA to synergistically enhance the activity of hemin.^[15]^ In this complex, G‐quadruplex, which as folded a high‐order structure composed of base‐stacked quartets, can serve as a supramolecular scaffold to stabilize hemin against dimerization in aqueous solution, and also provide an axial nucleobase ligand. The peptide can provide activating groups (e.g., histidine) at the distal side. These two components can be located at either side of hemin, resembling the three‐dimensional structure of the native hemin pocket. Therefore, in this work, we explored the effect of the methylation of the histidine on the catalysis of the Fmoc‐His/G‐quadruplex DNA (G‐DNA)/hemin complex (Figure S16). We find that adding G‐DNA can indeed enhance the activity of Fmoc‐_εm_His/hemin at various Fmoc‐_εm_His concentrations. However, similar to Fmoc‐_δm_His/hemin, the activity of Fmoc‐_δm_His/G‐DNA/hemin decreased markedly with increasing concentrations of Fmoc‐_δm_His, nearly reaching zero at 3 mM. The UV‐Vis spectra showed that the spectral characteristics of Fmoc‐_εm_His/G‐DNA/hemin, particularly within the 450–700 nm range, closely resembled those of G‐DNA/hemin (626 and 504 nm, with a weak absorbance at 530 nm), reflecting the preferential coordination of hemin iron to the nucleobase ligand of G‐DNA (Figure S17A). This suggests that the histidine residues may be located at the distal side of hemin to promote the catalytic activity. In comparison, the Fmoc‐_δm_His/G‐DNA/hemin complex exhibited spectral characteristics of Fmoc‐_δm_His/hemin (Figure S17B), confirming the robust interaction of Fmoc‐_δm_His with hemin, which may explain the absence of any observed catalytic synergy between Fmoc‐_δm_His and G‐DNA.

## 3 CONCLUSION

We have reported the self‐assembly of Fmoc‐His with hemin to produce the peroxidase‐mimetic catalyst, and explored the impact of the heterogeneity of the imidazole nitrogen of Fmoc‐His on hemin activity through histidine methylation. It was found that Fmoc‐_εm_His significantly enhanced the peroxidative activity of hemin, but Fmoc‐_δm_His/hemin was almost inactive. The experimental and theoretical results indicate that the stronger interaction between the imidazole nitrogen of Fmoc‐_δm_His and hemin iron, particularly at the distal side of hemin, along with the increased association of Fmoc‐_δm_His resulting in a higher possibility of its imidazole nitrogen binding to hemin, contributes to the challenge in substrate adsorption, H_2_O_2_, to the hemin iron. The H_2_O_2_ adsorption is a critical to the formation of Compound I intermediate, which is typically considered as the rate‐limiting step in the catalytic cycle of native peroxiase. Moreover, the _δm_His at the distal side of hemin can also potentially hinder the subsequent access of the reductive sustrate (e.g. TMB) to ferryl oxo species of Compound I and restrict the flexibility of the oxo needed for optimal catalytic reactions. Even after forming complexes with G‐quadruplex DNA, Fmoc‐_δm_His/DNA/hemin demonstrated significantly lower activity than Fmoc‐_εm_His/DNA/hemin and Fmoc‐His/DNA/hemin, although they all showed synergistic catalysis. This research may not fully explain why nature peroxidase selectively uses the nitrogen at ε or δ position of histidine, a phenomenon also influenced by surrounding amino acid residues and the rigidity of the three‐dimensional folding. However, this work offers insights into the favorable environment surrounding hemin and introduces a novel approach for designing histidine‐based supramolecular materials, which holds substantial promise in biotechnology, optoelectronic engineering, and biomedicine.

## AUTHOR CONTRIBUTIONS

Ruikai Du, Yunbo Lv and Haifeng Wu contributed equally to this work. Zhen‐Gang Wang conceived, designed, and supervised the experiments. Ruikai Du performed the experiments. Zhen‐Gang Wang, Ruikai Du and Yunbo Lv collected and analyzed the data. Haifeng Wu performed density functional theory. Baoli Zhang conducted hydrogen nuclear magnetic resonance. Yunbo Lv, Shichao Xu and Shan Li provided suggestions on the work. Zhen‐Gang Wang, Ruikai Du and Yunbo Lv wrote the manuscript. All authors have given approval to the final version of the manuscript.

## CONFLICT OF INTEREST STATEMENT

The authors declare no conflicts of interest.

## ETHICS STATEMENT

No animal or human experiments were involved in this study.

## Supporting information

Supporting Information S1

## Data Availability

The data that support the findings of this study are available in the supplementary material of this article.

## References

[1] a) Z. Chen , Y. Yu , Y. Gao , Z. Zhu , ACS Nano. 2023, 17, 13062. 37399457 10.1021/acsnano.3c04378

[2] a) Q. Liu , A. Kuzuya , Z.‐G. Wang , iScience 2023, 26, 105831. 36636357 10.1016/j.isci.2022.105831 PMC9830222

[3] K. J. Koebke , F. Yu , C. Van Stappen , T. B. J. Pinter , A. Deb , J. E. Penner‐Hahn , V. L. Pecoraro , J. Am. Chem. Soc. 2019, 141, 7765. 30983335 10.1021/jacs.9b00196 PMC6824201

[4] a) A. J. Burke , S. L. Lovelock , A. Frese , R. Crawshaw , M. Ortmayer , M. Dunstan , C. Levy , A. P. Green , Nature 2019, 570, 219. 31132786 10.1038/s41586-019-1262-8

[5] A. M. Azevedo , V. C. Martins , D. M. Prazeres , V. Vojinović , J. M. Cabral , L. P. Fonseca , Biotechnol. Annu. Rev. 2003, 9, 199. 14650928 10.1016/s1387-2656(03)09003-3

[6] a) A. Dominguez , A. Fernandez , N. Gonzalez , E. Iglesias , L. Montenegro , J. Chem. Educ. 1997, 74, 1227.

[7] V. Jayawarna , M. Ali , T. A. Jowitt , A. F. Miller , A. Saiani , J. E. Gough , R. V. Ulijn , Adv. Mater. 2006, 18, 611.

[8] a) S. Alavez , M. C. Vantipalli , D. J. S. Zucker , I. M. Klang , G. J. Lithgow , Nature 2011, 472, 226. 21451522 10.1038/nature09873 PMC3610427

[9] J. Silver , B. Lukas , Inorg. Chim. Acta. 1983, 78, 219.

[10] a) A. Lombardi , F. Nastri , V. Pavone , Chem. Rev. 2001, 101, 3165. 11710067 10.1021/cr000055j

[11] M. Andersson , J. Hedin , P. Johansson , J. Nordström , M. Nydén , J. Phys. Chem. A 2010, 114, 13146. 21114301 10.1021/jp1062868

[12] a) S. Engbers , Y. Guo , J. E. M. N. Klein , Angew. Chem. Int. Ed. 2023, 62, e202313006. 10.1002/anie.202313006 PMC10704252 37751302

[13] M. Tanokura , Biochim. Biophys. Acta 1983, 742, 576. 6838890 10.1016/0167-4838(83)90276-5

[14] a) F. Weigend , R. Ahlrichs , Phys. Chem. Chem. Phys. 2005, 7, 3297. 16240044 10.1039/b508541a

[15] a) Q. Liu , H. Wang , X. Shi , Z.‐G. Wang , B. Ding , ACS Nano 2017, 11, 7251. 28657711 10.1021/acsnano.7b03195

