# Supplementary material for: N‐methylation of histidine to tune tautomeric preferences in histidine‐heme coordination and enzyme‐mimetic catalysis
Source: Smart Mol. 2024 Jul 18;2(3):e20240012. doi: 10.1002/smo.20240012 (PMC12118220; doi:10.1002/smo.20240012)
Supplement: Supplementary file 1 — Supporting Information S1 [file SMO2-2-e20240012-s001.docx]

Supporting Information

**N-Methylation of Histidine to Tune Tautomeric Preferences in Histidine-Heme Coordination and Enzyme-Mimetic Catalysis**

Ruikai Du^‡^, Yunbo Lv^‡^, Haifeng Wu^‡^, Baoli Zhang,Yuanxi Liu, Shichao Xu, Shan Li, and Zhen-Gang Wang^*^

State Key Laboratory of Organic-Inorganic Composites, Key Lab of Biomedical Materials of Natural Macromolecules (Ministry of Education), Beijing Laboratory of Biomedical Materials, College of Materials Science and Engineering, Beijing University of Chemical Technology, Beijing 100029, China

‡ These authors contributed equally to this work

Corresponding author: [wangzg@buct.edu.cn](mailto:wangzg@buct.edu.cn)

[1. Experimental procedures 3](#_Toc169093583)

[1.1 Materials 3](#_Toc169093584)

[1.2 Instruments 3](#_Toc169093585)

[1.3 Activity assay 3](#_Toc169093586)

[1.4 Fluorescence spectra of pyrene 4](#_Toc169093587)

[1.5 Fluorescence spectra of thioflavin T 4](#_Toc169093588)

[1.6 Conformation search of Fmoc modified histidine derivative dimer 4](#_Toc169093589)

[1.7 Fluorescence assay 5](#_Toc169093590)

[2. Supplementary figures and tables 6](#_Toc169093591)

### 1. Experimental procedures

#### 1.1 Materials

Fmoc-Histidine-OH (Fmoc-His), Fmoc-1-Methyl-Histidine-OH (Fmoc-_εm_His) and Fmoc-3-Methyl-Histidine-OH (Fmoc-_δm_His) were purchased from Aladdin (China). Hemin was purchased from Alfa Aesar (China). 3,3′,5,5′-tetramethylbenzidine (TMB), 2,4-Dichlorophenol (2,4-DCP) and 4-Aminoantipyrine (4-AP) were purchased from Aladdin (China). G-quadruplex (G-DNA) was synthesized by HIPPO BIOTECHNOLOGY. Thioflavin (ThT, with purity level above 98%) was purchased from Beijing InnoChem Science & Technology Co., Ltd. Water was deionized using a Milli-Q system (≥18.25 MΩ·cm^–1^).

#### 1.2 Instruments

UV-Vis absorption spectra were recorded using a UV-2600 spectrometer equipped with a temperature-control accessory (Shimadzu). Fluorescent emission spectra were recorded using a G9800A fluorescence spectrophotometer with a temperature-control accessory (Agilent Technologies). ^1^H NMR spectra were recorded in DMSO-*d*_6_ on Bruker AV600 (600 MHz).

#### 1.3 Activity assay

The fluorenyl-modified histidine (or its N-methylated tautomer) was dissolved in ultrapure water to prepare 200 mM stock solutions. Subsequently, the required concentrations of fluorenyl-modified histidine (or its N-methylated tautomer) and hemin were added to 50 mM PB buffer (pH = 7.0). Using TMB and H_2_O_2_ (with a molar extinction coefficient of 39000 mol^-1^·L·cm^-1^ for the oxidized TMB), the reactions were conducted. Time-dependent absorbance changes were recorded, and this data was used to determine the initial catalytic velocity (*V*_i_) and apparent kinetic parameters.

#### 1.4 Fluorescence spectra of pyrene

To prepare the pyrene stock solution, 0.8 mg of pyrene was dissolved in 8 mL of methanol. For the fluorescence assay, the sample was mixed with the pyrene stock solution in a volume ratio of 100:1 immediately before analysis. The experimental conditions were set as follows: an excitation wavelength (λ_ex_) of 334 nm and an emission wavelength (λ_em_) ranging from 350 nm to 450 nm. Two distinct fluorescent peaks, labeled as I_1_ and I_3_, were observed at 373 nm and 384 nm, respectively. The intensity ratio (I_1_/I_3_) between these two peaks can be utilized to investigate the polarity of the microenvironments within the amino acid amphiphiles.

#### 1.5 Fluorescence spectra of thioflavin T

For the fluorescence assay, the sample was mixed with the thioflavin T stock solution in a volume ratio of 100:1 immediately before analysis. The experimental conditions were set as follows: an excitation wavelength (λ_ex_) of 450 nm and an emission wavelength (λ_em_) ranging from 470 nm to 600 nm. The intensity of 490 nm can be utilized to investigate the polarity of the microenvironments within the amino acid amphiphiles.

#### 1.6 Conformation search of Fmoc modified histidine derivative dimer

The conformational search of the dimer was first performed with molecular dynamics sampling through the xTB program.^[1]^ A 100 ps simulation was performed at 300 K with a step size of 1 fs and sampling every 50 fs for a total of 2000 frames. For the results of MD sampling, we used the Crest program to perform structural optimization and deduplication at the GFN0-xTB and GFN2-xTB levels with implicit solvent models.^[2]^ The deduplication step is completed through the isostat plug-in of the Molclus program.^[3]^ Finally, several conformers with cutoff energies of 5 kcal/mol were selected for geometric optimization and single point calculations under the DFT method. The geometry optimizations were performed with the B3LYP functional^[4]^ with the Becke-Johnson damping scheme (D3BJ)^[5]^, and the 6-31G(d,p) basis set^[6]^ were using for all atoms. The IEFPCM solvent model^[7]^ of water is used during geometry optimization. The single point energy calculations were performed using the M06-2X functional^[8]^ with Grimme dispersion corrections (GD3), and the def2-TZVP basis set^[9]^ was using for all atoms. SMD solvent model^[10]^ of water is used during geometry optimization. The Boltzmann population of the conformations was solved by the partition function. Binding energy with BSSE correction using counterpoise method was performed with the ma-TZVP basis set.^[11]^

#### 1.7 Fluorescence assay

The amino acid amphiphiles were dissolved in ultrapure water to prepare 100 mM stock solutions. Subsequently, the required concentrations of amino acid amphiphiles were added to ultrapure water. The experimental conditions involved an excitation wavelength of 290 nm and an emission wavelength ranging from 300 nm to 550 nm, as well as an excitation wavelength of 380 nm and an emission wavelength ranging from 390 nm to 550 nm. The scan rate was set at 120 nm/min.

### 2. Supplementary figures and tables

**Figure S1.** Active site structures of different enzymes. (A) Catechol oxidase (PDB: 1BT3), (B) Peroxidases (PDB: 1W4W), and (C) Carbonic anhydrases (PDB: 3D92).


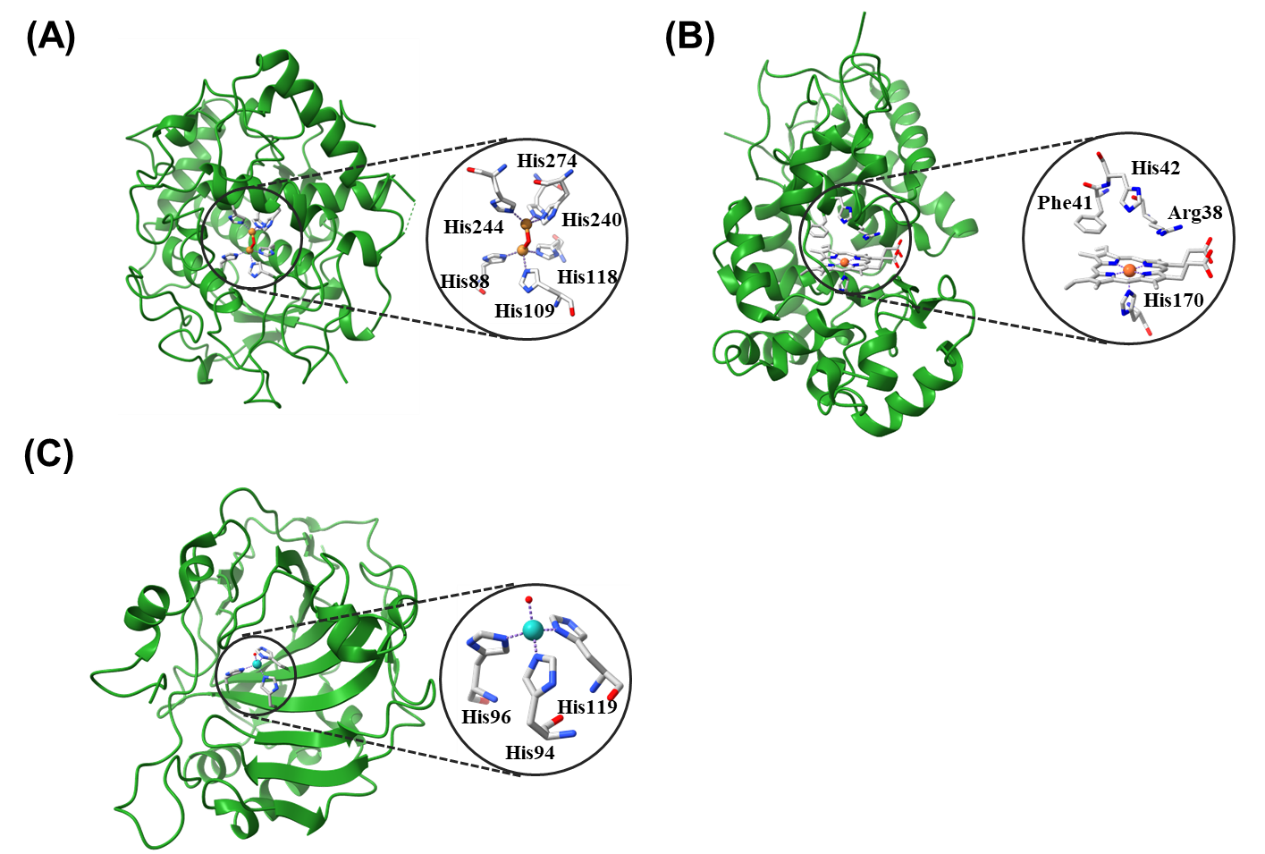


**Figure S2** The initial velocity of catalytic oxidation of TMB by Fmoc-His/hemin assembly. [hemin] = 0.5 μM, [TMB] = 0.3 mM, [H_2_O_2_] = 1 mM.

**Figure S3.** The initial velocity of catalytic oxidation of TMB by Fmoc-His/hemin assembly at different H_2_O_2_ concentrations. [Fmoc-His] = 3 mM, [hemin] = 0.5 μM, [TMB] = 0.3 mM.

**Figure S4.** The initial velocity of catalytic oxidation of TMB by (A) Fmoc-_εm_His/hemin, (B) Fmoc-_δm_His/hemin and (C) Fmoc-His/hemin assemblies at different TMB concentrations. [Fmoc-_εm_His] = 3 mM, [Fmoc-_δm_His] = 3 mM, [hemin] = 0.5 μM, [H_2_O_2_] = 15 mM.


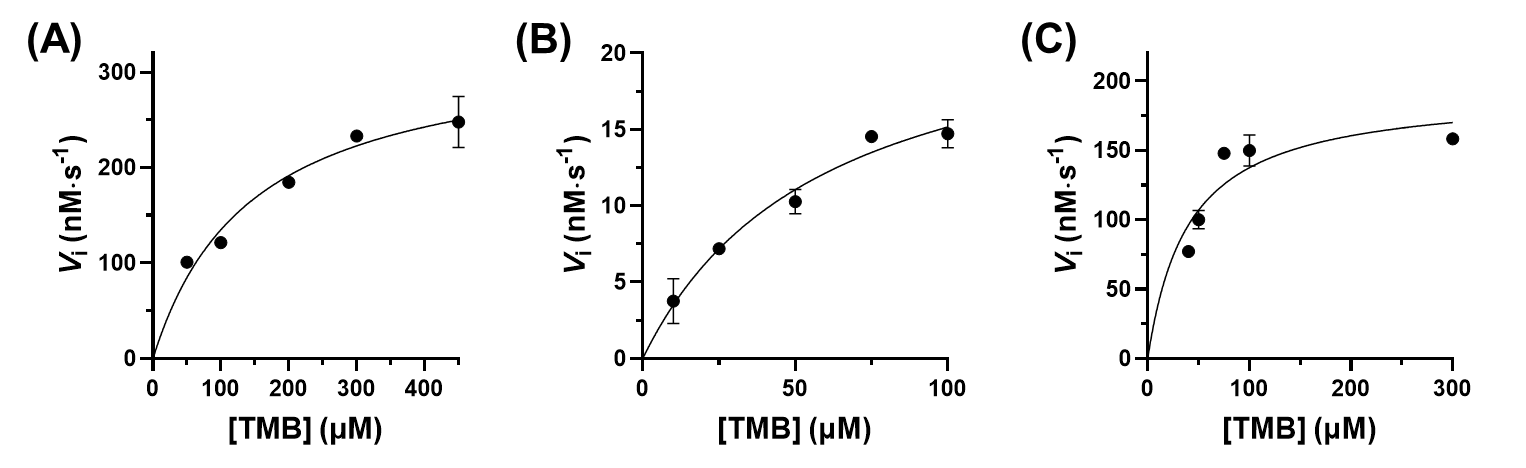


**Figure S5.** Arrhenius plots for (A) Fmoc-_εm_His/hemin and (B) Fmoc-_δm_His/hemin assemblies. [Fmoc-_εm_His] = 3 mM, [Fmoc-_δm_His] = 3 mM, [hemin] = 0.5 μM, [TMB] = 0.3 mM, [H_2_O_2_] = 1 mM.


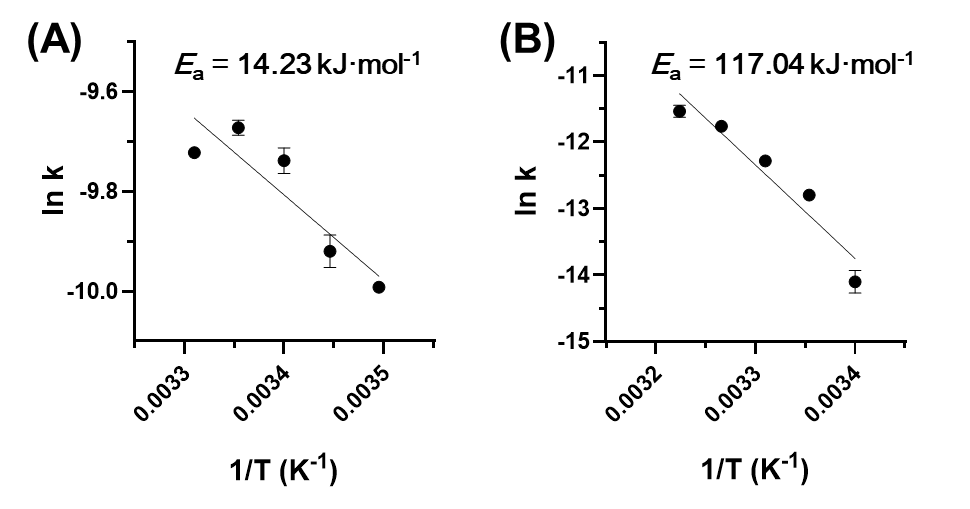


**Figure S6.** (A) The initial velocity of oxidation of 2,4-DCP catalyzed by Fmoc-His/hemin, Fmoc-_εm_His/hemin and Fmoc-_δm_His/hemin assemblies. (B) The initial velocity of oxidation of ABTS catalyzed by Fmoc- H/hemin, Fmoc-_εm_His/hemin and Fmoc-_δm_His/hemin assemblies. [Fmoc-His] = 3 mM, [Fmoc-_εm_His] = 3 mM, [Fmoc-_δm_His] = 3 mM, [hemin] = 0.5 μM, [2,4-DCP] = 0.3 mM, [4-AP] = 0.3 mM, [ABTS] = 0.3 mM, [TMB] = 0.3 mM, [H_2_O_2_] = 1 mM.


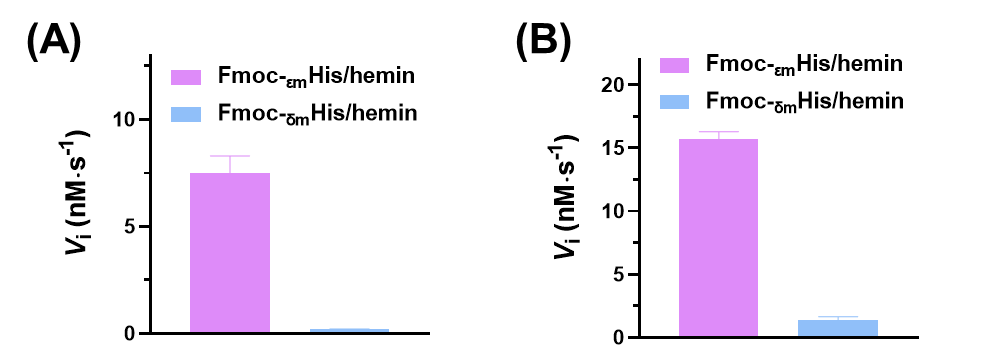


**Figure S7.** Fluorescence spectra of pyrene in (A) Fmoc-_εm_His and (B) Fmoc-_δm_His. (C) I_1_/I_3_ of pyrene in Fmoc-_εm_His and Fmoc-_δm_His at different concentrations. [pyrene] = 4 μM.


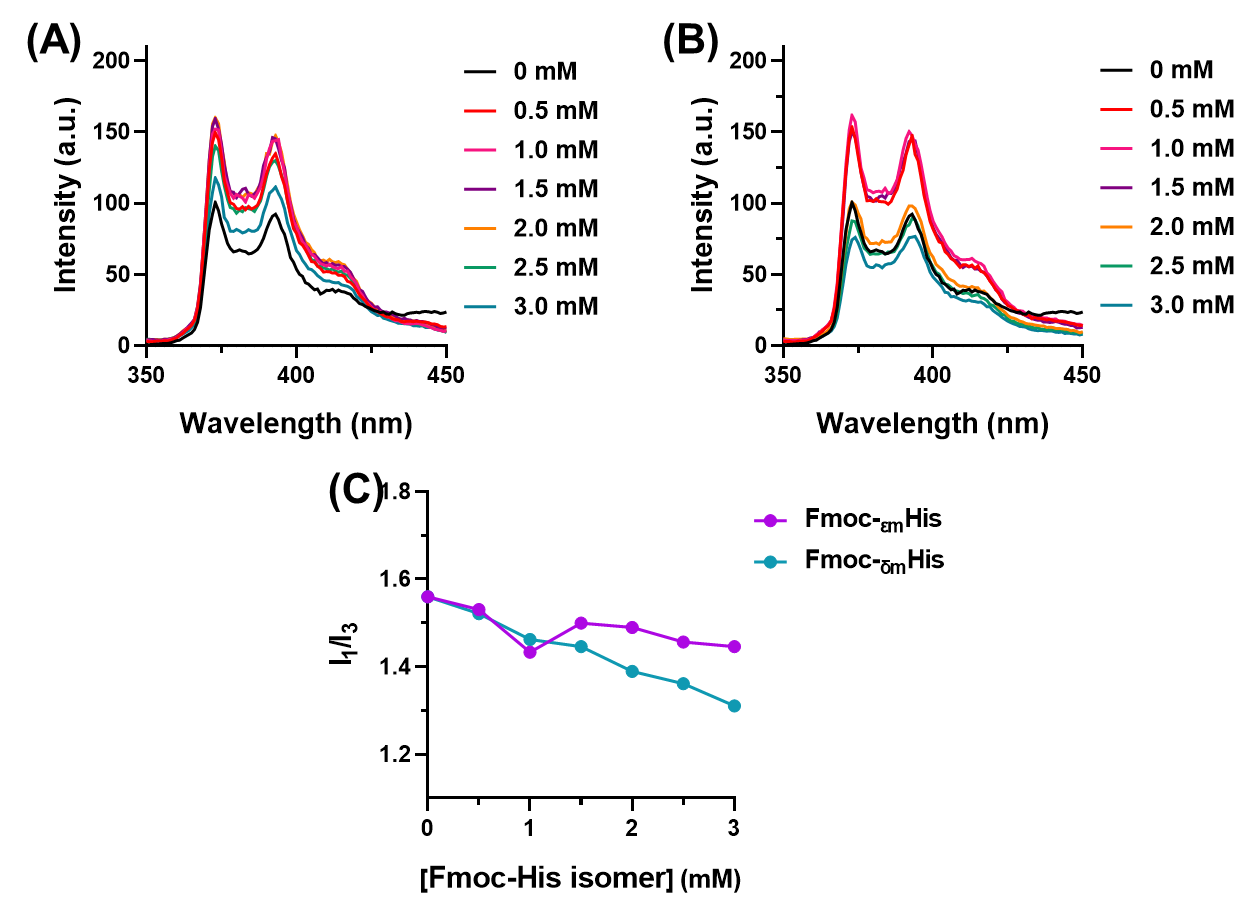


**Figure S8.** Fluorescence spectra of the Fmoc-His assembly.

**Figure S9.** Fluorescence spectra of thioflavin T (ThT) in (A) Fmoc-_εm_His and (B) Fmoc-_δm_His. [ThT] = 100 μM.


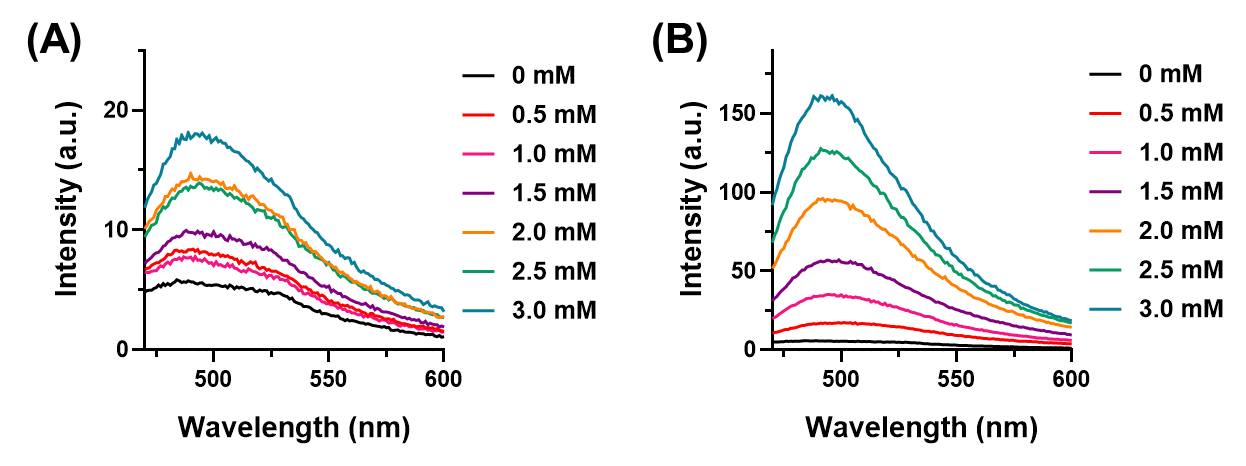


**Figure S10.** Schematic diagram of the spatial location of the Fmoc-_εm_His dimers.


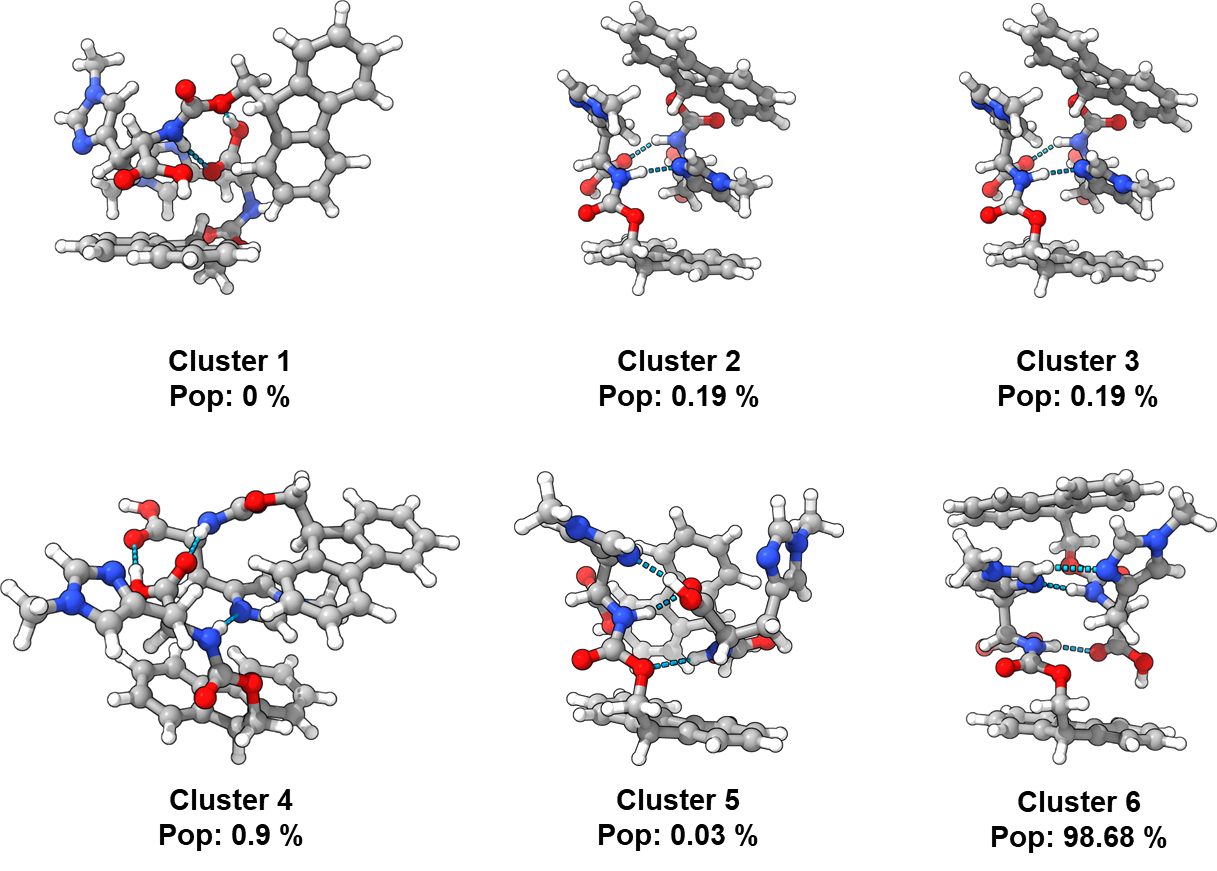


**Figure S11.** Theoretical model of the coordination of Hie with hemin iron. In the chemical structures, nitrogen atoms are depicted in blue, oxygen atoms in red, iron atoms in brown, carbon atoms in dark gray, and hydrogen atoms in light gray.


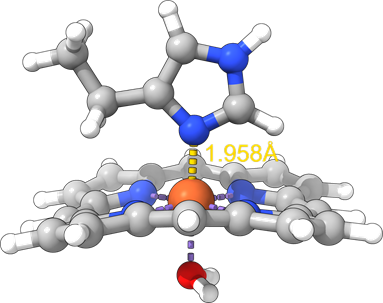


**Figure S12.** Theoretical model of the coordination of Hid with hemin iron. In the chemical structures, nitrogen atoms are depicted in blue, oxygen atoms in red, iron atoms in brown, carbon atoms in dark gray, and hydrogen atoms in light gray.


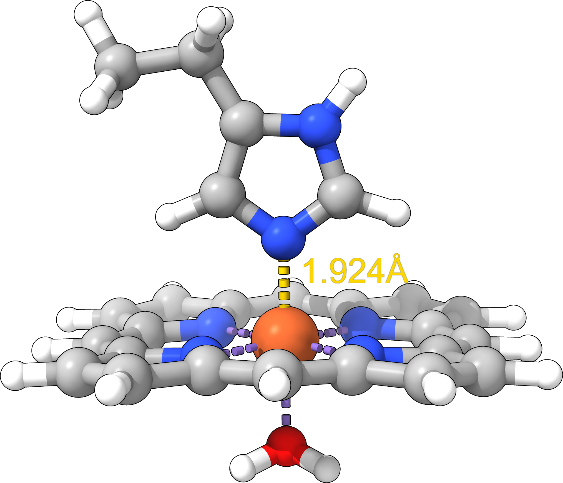


**Figure S13** Binding energy of Hie, Hid, _εm_His and _δm_His.

**Figure S14.** Electrostatic potential of Hie, Hid, _εm_His and _δm_His.


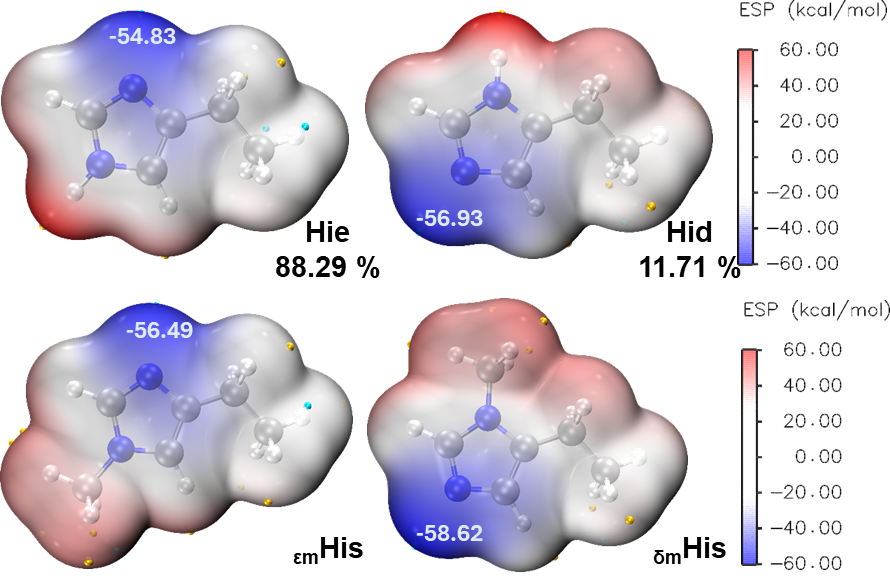


**Figure S15.** The initial velocity of oxidation of 2,4-DCP catalyzed by Fmoc-_εm_His/hemin and Fmoc-_δm_His/hemin at different temperatures.

**Figure S16.** The initial velocity of catalytic oxidation of TMB by Fmoc-His/G-DNA/hemin, Fmoc-_εm_His/G-DNA/hemin, and Fmoc-_δm_His/G-DNA/hemin assemblies. [hemin] = 0.5 μM, [TMB] = 0.3 mM, [H_2_O_2_] = 1 mM.

**Figure S17.** UV-Vis spectra of (A) Fmoc-_εm_His/G-DNA/hemin and (B) Fmoc-_δm_His/G-DNA/hemin. [hemin] = 2 μM.


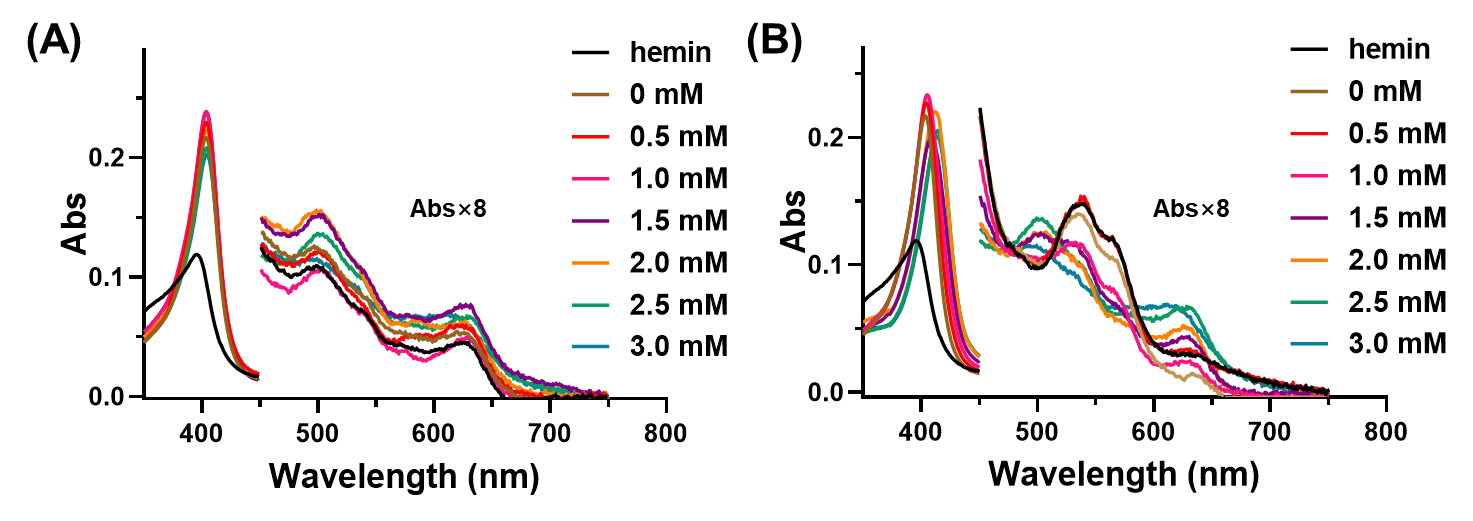


**Table S1.** Apparent kinetic parameters for the hemin complex with respect to TMB oxidation.^[12]^

| **Samples** | **Substrate** | ***k*_cat_**  **(s^-1^)** | ***K*_m_**  **(mM)** | ***k*_cat_/*K*_m_**  **(s^-1^·M^-1^)** | **Ref.** |
| --- | --- | --- | --- | --- | --- |
| Fmoc-_εm_His/hemin | TMB | 0.661 | 0.1449 | 4561 | This work |
| Fmoc-_δm_His/hemin | TMB | 0.048 | 0.0496 | 967.7 | This work |
| LMLHLFL/hemin | TMB | 0.99 | 0.106 | 9339 | Ref.12a |
| CCA-YH | TMB | 0.0197 | 0.584 | 33.8 | Ref.12b |
| C_16_-AHL_3_K_3_-CO_2_H/hemin | TMB | 0.046 | 0.0294 | 1564 | Ref.12c |
| C_16_-MHL_3_K_3_-CO_2_H/hemin | TMB | 0.037 | 0.0172 | 2150 | Ref.12c |
| KH/hemin | TMB | 0.047 | 0.070 | 671 | Ref.12d |
| NHNH/hemin | TMB | 0.099 | 0.17 | 582 | Ref.12d |
| NHNHNH/hemin | TMB | 0.063 | 0.099 | 636 | Ref.12d |
| G5HH | TMB | 0.13 | 0.1368 | 950 | Ref.12e |
| PS2.M | TMB | 0.32 | 0.0529 | 6049 | Ref.12e |

**Supplementary references**

[1] a) C. Bannwarth, E. Caldeweyher, S. Ehlert, A. Hansen, P. Pracht, J. Seibert, S. Spicher, S. Grimme, *WIREs Comput. Mol. Sci.* **2020**, *11*, e01493; b) S. Grimme, C. Bannwarth, P. Shushkov, *J. Chem. Theory Comput*. **2017**, *13*, 1989; c) C. Bannwarth, S. Ehlert, S. Grimme, *J. Chem. Theory Comput*. **2019**, *15*, 1652; d) P. Pracht, E. Caldeweyher, S. Ehlert, S. Grimme, *(Preprint)* DOI: 10.26434/chemrxiv.8326202.v1, version 1, *ChemRxiv*. June, **2019**.

[2] P. Pracht, F. Bohle, S. Grimme, *Phys. Chem. Chem. Phys*. **2020**, *22*, 7169.

[3] Tian Lu, Molclus program, <http://www.keinsci.com/research/molclus.html>, version 1.12, Aug, **2023**.

[4] A. D. Becke, *J. Chem. Phys.* **1993**, *7*, 5648.

[5] a) S. Grimme, S. Ehrlich, L. Goerigk, *J. Comput. Chem.* **2011**, *32*, 1456; b) S. Grimme, J. Antony, S. Ehrlich, H. Krieg, *J. Chem. Phys.* **2010,** *132*, 154104.

[6] W. J. Hehre, R. Ditchfield, J. A. Pople, *J. Chem. Phys.* **1972,** *56*, 2257.

[7] a) E. Mennucci, B. Tomasi, *J. Chem. Phys.* **1997**, *107*, 3032; b) M. Cossi, V. Barone, B. Mennucci, J. Tomasi, *Chem. Phys. Lett.* **1998**, *286*, 253; c) B. Mennucci, J. Tomasi, *J. Chem. Phys.* **1997**, *106*, 5151.

[8] Y. Zhao, D. G. Truhlar, *Theor. Chem. Acc.* **2008**, *120*, 215.

[9] a) F. Weigend, R. Ahlrichs, *Phys.* *Chem. Chem. Phys*. **2005**, *7*, 3297; b) J. Zheng, X. Xu, D. G. Truhlar, *Theor. Chem. Acc.* **2011**, *128*, 295.

[10] A. V. Marenich, C. J. Cramer, D. G. Truhlar, *J. Phys. Chem. B* **2009**, *113*, 6378.

[11] J. Zheng, X. Xu, D. G. Truhlar, *Theor. Chem. Acc.* **2011**, *128*, 295.

[12] a) O. Zozulia, L. R. Marshall, I. Kim, E. M. Kohn, I. V. Korendovych, *Chem. Eur. J.* **2021**, *27*, 5388; b) Z. Ma, L. Yang, Y. Wang, M. Wang, W. Qi, Z. He, *Chem. Eng. J.* **2021**, *416*, 129149. c) L. A. Solomon, J. B. Kronenberg, H. C. Fry, *J. Am. Chem. Soc.* **2017**, *139*, 8497; d) Y. Zhang, X. Li, *J. Mater. Chem. B* **2023**, *11*, 3898; e) E. Shokri, M. Hosseini, M. N. Boldaji, K. Shahsavar, A. amiri-Sadeghan, N. Nasiri, A. Bahmani, M. R. Ganjali, A. A. Saboury, *Mol. Catal* **2022**, *519*, 112156.
